# Yolk protein is expressed in the insect testis and interacts with sperm

**DOI:** 10.1186/1471-213X-8-64

**Published:** 2008-06-13

**Authors:** Piotr Bebas, Joanna Kotwica, Ewa Joachimiak, Jadwiga M Giebultowicz

**Affiliations:** 1 Department of Animal Physiology, Faculty of Biology, University of Warsaw, Warsaw, Poland; 2 Department of Zoology, Oregon State University, Corvallis, OR, USA

## Abstract

**Background:**

Male and female gametes follow diverse developmental pathways dictated by their distinct roles in fertilization. While oocytes of oviparous animals accumulate yolk in the cytoplasm, spermatozoa slough off most of their cytoplasm in the process of individualization. Mammalian spermatozoa released from the testis undergo extensive modifications in the seminal ducts involving a variety of glycoproteins. Ultrastructural studies suggest that glycoproteins are involved in sperm maturation in insects; however, their characterization at the molecular level is lacking. We reported previously that the circadian clock controls sperm release and maturation in several insect species. In the moth, *Spodoptera littoralis*, the secretion of glycoproteins into the seminal fluid occurs in a daily rhythmic pattern. The purpose of this study was to characterize seminal fluid glycoproteins in this species and elucidate their role in the process of sperm maturation.

**Results:**

We collected seminal fluid proteins from males before and after daily sperm release. These samples were separated by 2-D gel electrophoresis, and gels were treated with a glycoprotein-detecting probe. We observed a group of abundant glycoproteins in the sample collected after sperm release, which was absent in the sample collected before sperm release. Sequencing of these glycoproteins by mass spectroscopy revealed peptides bearing homology with components of yolk, which is known to accumulate in developing oocytes. This unexpected result was confirmed by Western blotting demonstrating that seminal fluid contains protein immunoreactive to antibody against yolk protein YP2 produced in the follicle cells surrounding developing oocytes. We cloned the fragment of *yp2 *cDNA from *S. littoralis *and determined that it is expressed in both ovaries and testes. *yp2 *mRNA and YP2 protein were detected in the somatic cyst cells enveloping sperm inside the testis. During the period of sperm release, YP2 protein appears in the seminal fluid and forms an external coat on spermatozoa.

**Conclusion:**

One of the yolk protein precursors YP2, which in females accumulate in the oocytes to provision developing embryos, appears to have a second male-specific role. It is produced in the testes and released into the seminal fluid where it interacts with sperm. These data reveal unexpected common factor in the maturation of insect eggs and sperm.

## Background

Germ-line cells of male and female undergo a complicated process of maturation before they become fertilization-competent spermatozoa or oocytes, respectively. At the earliest stages of their development, germ-line stem cells become enveloped by somatic cells; the two cell types interact extensively throughout gamete maturation. In insects, cells surrounding developing oocyte are called the follicle cells, while cells enveloping clones of differentiating spermatozoa are called the cyst cells.

Follicle cells have multiple functions; they protect and nourish the growing oocyte, control chorion formation, and are involved in the spatial patterning of the egg [1]. Follicle cells also play important roles in vitellogenesis. They produce yolk proteins (YP) precursors, which accumulate in yolk spheres of maturing oocytes. In addition, follicle cells control the uptake by the oocyte of yolk proteins (vitellogenins) produced by the fat body [2].

Compared to follicle cells, the functions of their somatic equivalents in insect testes, the cyst cells, are poorly understood. The cyst cells surround each spermatogonial founder cell and continue to envelope differentiating spermatocytes and elongating spermatids [3,4]. In higher insects, developing spermatozoa are aligned in sperm bundles [5]. During the release from the testis, spermatozoa are freed from the cyst cells; the latter appear to undergo fragmentation and phagocytosis by the adjacent testis epithelium [6-8].

Spermatozoa released from testis undergo further maturation in order to acquire fertilizing capacity. The process of extra-testicular maturation of sperm has been studied extensively in mammals. It has been found that many glycoproteins released from the Sertoli cells and from the reproductive tract epithelia participate in sperm maturation and contribute to the extracellular coat that is formed on spermatozoa [9-11]. Ultrastructural studies on several moth species suggested that insect spermatozoa acquire an extracellular coat after the release from testis [12,13]; however, the coat components have not been identified biochemically. The moth *Spodoptera littoralis *is especially well suited to study extra-testicular sperm maturation because males of this species release several hundreds of sperm bundles per day from the testis into the upper vas deferens (UVD) [14]. The release of sperm in this and other moths is controlled by the circadian clock and occurs within a few hours after the onset of dark phase in the 16 h light: 8 h dark photoperiod [14,15]. The release of sperm into the UVD coincides with circadian clock-controlled acidification of the seminal fluid and the secretion of glycoprotein rich granules into the UVD [16,17]. The goal of the current study was to identify glycoproteins in the UVD seminal fluid at the time of sperm release. Surprisingly, peptides derived from the most prominent glycoprotein showed homology to female yolk components from several moth species. Further, the antibody against yolk protein YP2, which is produced in the female follicle cells [18,19] immuno-detected a single protein in the male seminal fluid. We then cloned the fragment of *yp2 *cDNA from *S. littoralis *and determined that it is expressed in both ovaries and testes. We present evidence that YP2 protein is produced in the male cyst cells and interacts with spermatozoa released into the UVD lumen.

## Results

### Seminal fluid of *S. littoralis *contains glycoproteins with homology to yolk proteins

**Figure 1:**
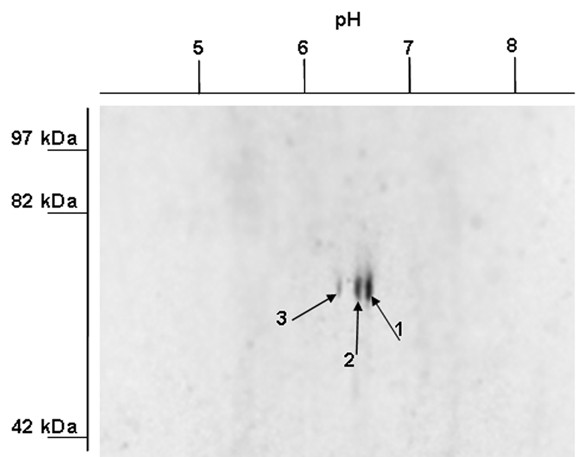
**Glycoprotein detection in the UVD seminal fluid of *Spodoptera littoralis***. Proteins obtained from the UVD lumen were separated by 2-D gel electrophoresis and probed with Pro-Q Emerald 300 Glycoprotein Gel Stain. Black arrows indicate most abundant glycoprotein spots with isoelectric point (pI) between 6 and 7 (numbered 1–3). These proteins were submitted to mass spectrometry analysis, which detected in proteins #1 and #2 several peptides with homology to proteins that form yolk in the oocytes.

Sperm bundles that are released from the moth testis into the lumen of the upper vas deferens (UVD) are bathed in seminal fluid. We reported previously that – coincident with sperm release – several glycoproteins are secreted into the UVD seminal fluid of the moth *S. littoralis *[17]. To characterize seminal fluid proteins, the UVDs were dissected from males 4 h after lights-on (day sample, prior to sperm release) and 4 h after lights-off (night sample, after sperm release from testis). Content of the UVD lumens from day and night samples were collected and sperm present in the night sample was separated from the seminal fluid by gentle centrifugation. Proteins contained in the seminal fluid were resolved by 2-D gel electrophoresis and the gel was incubated with a glycoprotein detecting stain. The stain revealed a prominent group of three proteins with molecular weight of approximately 75 kDa in the night sample that were absent in the day sample. (Figure 1, arrows 1–3). The three protein spots were excised from the gel and digested into peptides, which were analyzed by Q-TOF mass spectrometry combined with the nano-HPLC system. Protein spots No. 1 and 2 yielded five peptides homologous to yolk constituents from several species of Lepidoptera, while spot No. 3 yielded no such peptides.

**Figure 2:**
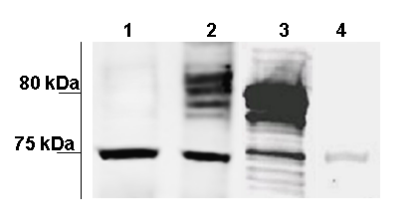
**Yolk protein cross-reactive material is present in the male reproductive tract**. Western blot with antiserum against YP2 from *P. interpunctella *detected a single protein band at ~75 kDa in seminal fluid of *S. littoralis *(Lane 1), and multiple bands at 75 – 80 kDa in extracts from *P. interpunctella *testis-UVD complexes (Lane 2) and ovarioles (Lane 3). The signal in *S. littoralis *seminal fluid was reduced when antibody was preabsorbed with *P. interpunctella *ovarian tissues prior to Western blotting (Lane 4). Equal amounts of total protein extracts (1 OD) were loaded in each lane.

To verify that yolk-related material is indeed present in the seminal fluid, proteins from the UVD lumen were separated by 1-D electrophoresis, transferred to the membrane, and probed with available antibodies made against various insect yolk proteins. We tested antibodies recognizing individual yolk proteins YP1, YP2, YP3 and YP4 from the moth, *Plodia interpunctella *[18], and antibody recognizing the three yolk peptides (YP1, YP2, and YP3) from *Drosophila melanogaster *[20]. Of these antibodies, only the one specifically recognizing YP2 of *P. interpunctella *[18] immunoreacted with a protein of approximately 75 kDa in seminal fluid of *S. littoralis *(Figure 2). This antibody stained protein band of similar size in both the ovary and the testis-UVD complex of *P. interpunctella*; additional staining was detected at approximately 80 kDa in these organs (Figure 2). Immunostaining in *S. littoralis *seminal fluid was drastically reduced when blot was probed withYP2 antibody preabsorbed with fixed *P. interpunctella *ovaries, suggesting that seminal fluid of *S. littoralis *indeed contains YP2 immunoreactive material (Figure 2).

### YP2 is expressed in *S. littoralis *ovary and accumulates in oocytes

**Figure 3:**
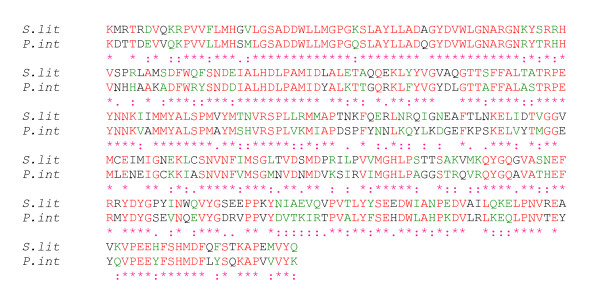
**YP2 gene is expressed in the ovary of *S. littoralis***. Alignment of *Spodoptera littoralis *(*S. lit*) predicted amino acid sequence with *Plodia interpunctella *(*P. int*) YP2 protein fragment. Identical amino acids are shown in red and marked by *, strongly similar amino acids are in green and marked by a colon, while weekly similar amino acids are marked by a dot. The two protein fragments show 59% identity and 85% similarity.

To determine whether YP2-like immunoreactive material corresponds to *S. littoralis *YP2 protein, we have undertaken the cDNA cloning of YP2 homolog from this species. We designed a series of degenerate primers deduced from highly conserved regions of YP2 from *P. interpunctella *and two other moth species, *Galleria mellonella *and *Bombyx mori*. A 240-bp cDNA product was amplified from total RNA extracted from *S. littoralis *ovaries by RT-PCR. After cloning and sequencing, the fragment was extended to the 3' region by 3'RACE-PCR. This resulted in identification of 1201 bp long cDNA product. It was sequenced and submitted to the database [GenBank:EU368829]. The deduced amino acid sequence showed high homology (59% identity, 85% similarity) to YP2 protein from *P. interpunctella *[21] when aligned with the ClustalW software [22] (Figure 3). Additional comparison of *S. littoralis *sequence with all three known YP2 homologs (see above) revealed 31% identity and 64% similarity among them.

The obtained cDNA sequence of *S. littoralis *was used to design the specific primers for amplification of a 660 bp cDNA product. Using these primers, we obtained cDNA of expected size from both ovaries and testis-vas deferens complexes of *S. littoralis*, but not from other tissues of males and females (not shown).

**Figure 4:**
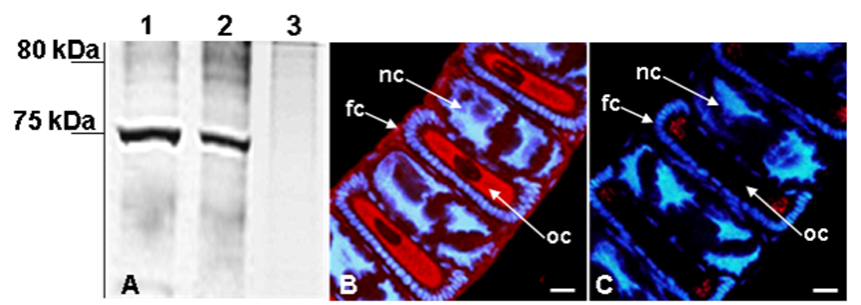
**YP2 protein contributes to yolk formation in the ovary of *S. littoralis***. **(A) **Western blot probed with YP2 antibody detected protein band at ~75 kDa in both seminal fluid (Lane 1) and ovarioles (Lane 2) of *S. littoralis*. Extracts of female fat body were immuno-negative (Lane 3). Equal amounts of total protein extracts (1 OD) were loaded in each lane. **(B) **Immunostaining of vitellogenic ovarioles of *S. littoralis *with YP2 antibody. Confocal microscopy detected staining in the oocyte cytoplasm (oc) and follicular cells (fc) that surround developing oocyte, but not in nurse cells (nc). **(C) **The YP2 signal was dramatically reduced when ovarian tissues were treated with antibody preabsorbed with fixed ovaries of *P. interpunctella*. Cell nuclei (blue) were stained with Hoechst 33258. Scale bar, 20 μm.

In the next experiment, we asked whether YP2 functions as yolk protein in *S. littoralis *females, similar to *P. interpunctella *[18]. Western blotting with YP2 antibody detected single protein band in the ovarian extracts but not in the fat body of *S. littoralis *females. The ovarian protein was of the same size as the immuno-positive protein in the seminal fluid (Figure 4A). YP2 antibody was then used to stain *S. littoralis *ovarioles containing maturing oocytes. Strong and specific YP2 signal was detected in vitellogenic oocytes and in the follicle cells (Figure 4B–C). These data demonstrate that the YP2 protein, which we first identified in male seminal fluid, is a yolk constituent in *S. littoralis *females. Since YP2 was not detected in the fat body (Figure 4A), it appears to be produced only in the follicle cells surrounding vitellogenic oocytes, similar to *P. interpunctella *[19].

### Distribution of YP2 protein and mRNA in the male reproductive system

**Figure 5:**
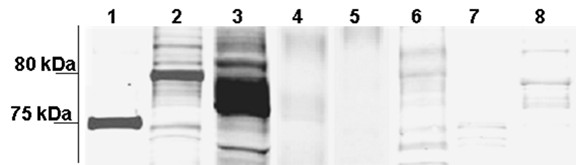
**Expression of YP2 protein in males of *S. littoralis***. Western blot of different segments of male reproductive system and non-reproductive organs probed with YP2 antibody. Single protein band at ~75 kDa was detected in the UVD seminal fluid (Lane 1) and band at ~80 kDa was detected in extracts of UVD wall (Lane 2). Abundant immuno-positive material with molecular weight between 75–80 kDa was detected in the extracts of whole testes (Lane 3). YP2 antibody did not give substantial immuno-positive signal in the lumen of seminal vesicles (SV, Lane 4), extracts of SV wall (Lane 5), hemolymph (Lane 6), flight muscles (Lane 7) or fat body (Lane 8). Equal amounts of total protein extracts (1 OD) were loaded in each lane.

The UVD seminal fluid contains proteins from two sources: the testis and the UVD secretory epithelium. To determine the origin of YP2 protein in the seminal fluid, we probed homogenates of the testes and the UVD wall with YP2 antibody by Western blot. In addition, we probed segments of the reproductive tract posterior to the UVD: seminal vesicles (SV), sperm-storing duplex, and ejaculatory duct. We also tested hemolymph, flight muscles and fat body for the presence of YP2. A single immunoreactive protein band of approximately 75 kDa was again detected in the extracts of the UVD seminal fluid (Figure 5). Extracts of the UVD wall contained immuno-positive band of approximately 80 kDa. Strong immunoreactivity with molecular weight between 75 and 80 kDa was observed in the extract of whole testes. We did not detect substantial YP2-immunopositive material in the seminal vesicles, hemolymph, muscles or the fat body (Figure 5), sperm-storing duplex, or ejaculatory duct (data not shown).

**Figure 6:**
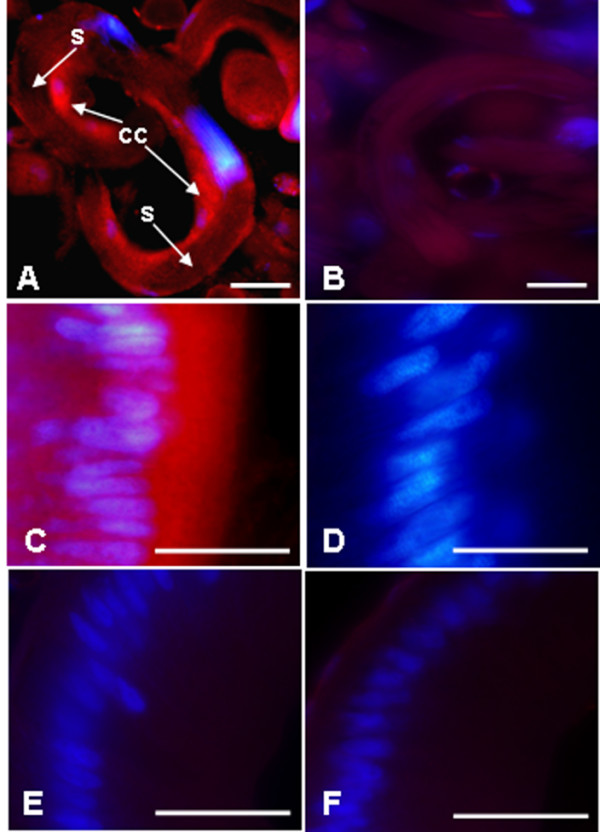
**Localization of YP2 protein in the *S. littoralis *male reproductive system by ICC**. **(A) **Strong signal was detected in the cyst cells (cc) surrounding sperm bundles in the testes but not in spermatozoa (s) inside the bundles. **(C) **Diffused YP2 immunostaining was also detected in the columnar epithelium of the UVD wall, but not in epithelium of the seminal vesicle **(E)**. Preabsorption of antibody with *P. interpunctella *ovarioles reduced staining in both cyst cells **(B) **and the UVD **(D) **with no effect on the seminal vesicle **(F)**.

Spatial distribution of YP2-immunopositive material in the reproductive system was determined by immunocytochemistry (ICC) with YP2 antibody on sections of the testis-vas deferens complexes. YP2 stained strongly the cytoplasm of cyst cells enclosing sperm bundles (Figure 6). Like other Lepidoptera, *S. littoralis *produce nucleated and anucleated sperm [23]. Cyst cells enveloping both types of sperm bundles stained specifically with YP2, while spermatozoa inside cysts appeared unstained (Figure 6 A-B). In addition to testicular cyst cells, sections of the UVD epithelial wall showed diffused but specific staining with YP2 antibody (Figure 6C–D). In contrast, seminal vesicle wall was immuno-negative (Figure 6E–F) in agreement with the results of Western blot (Figure 5, Line 5).

**Figure 7:**
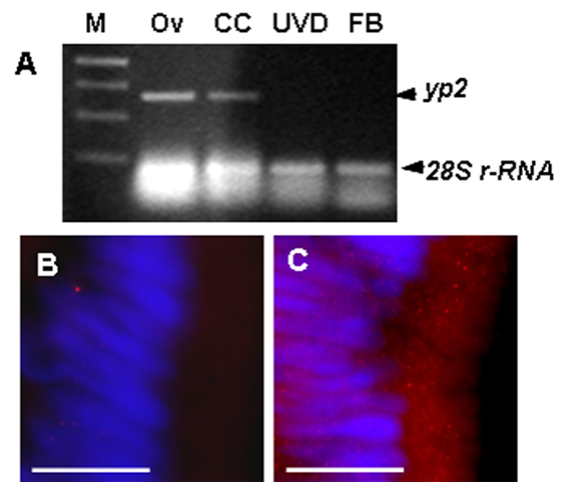
**YP2 is produced in the cyst cells**. **(A) **Total RNA was extracted separately from ovaries, testicular sperm bundles, UVD, and male fat body. RT-PCR was performed using *yp2*-specific primers; *28S r-RNA *gene was amplified as RNA loading control. A 660 nt PCR product representing *yp2 *was obtained from ovary (Ov, control) and from cyst cell-enclosed sperm bundles (CC) but not from the UVD epithelial cells or fat body (FB). M – markers. **(B) **No YP2 staining was observed in the UVD epithelium after the ligature was applied between testis and the UVD. **(C) **YP2 protein was detected in the UVDs of control sham-operated males. In all preparations nuclei were stained blue with Hoechst 33258. Bar = 20 μm.

Having detected YP2-like material in both the cyst cells and the UVD epithelial cells, we asked whether the *yp2 *gene is expressed in both cell types. Total RNA was extracted separately from cyst cell-enclosed testicular sperm bundles, UVD wall, and ovaries (serving as control). RT-PCR reactions were performed using *yp2*-specific primers. A PCR product of the expected size (660 nt) was obtained from ovary and from testicular sperm bundles but not from the UVD epithelial wall (Figure 7A). This data suggests that *yp2 *gene is not expressed in the UVD cells. To determine the origin of YP2-immunopositive material in the UVD epithelial cells, a thin silk thread was tied between the testes and the UVD preventing the release of sperm and other material from the testes. Reproductive systems from experimental and control sham-operated males were dissected 8 hours after treatment, fixed, sectioned, and stained with YP2 antibody. YP2 immunostaining was present in the epithelial cells of control UVDs that remained connected to testis. In contrast, UVDs that were isolated from the testis showed no YP2 signal in the epithelial cells (Figure 7B–C).

### YP2 protein coats the surface of spermatozoa

**Figure 8:**
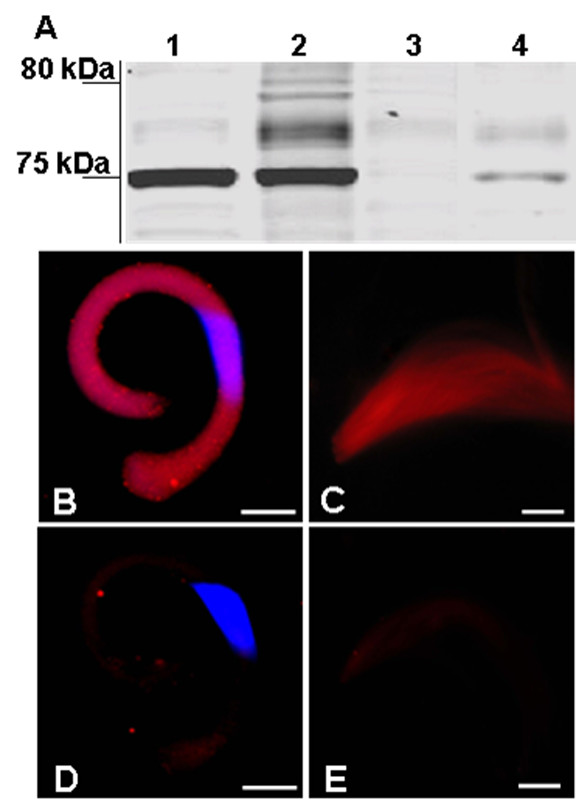
**YP2 protein forms transient extracellular coat on spermatozoa**. **(A) **Western blot detected YP2 immunoreactivity in both the UVD seminal fluid (Lane 1) and the UVD sperm (Lane 2). After sperm fraction was treated with 1 M NaCl, YP2 signal was absent from sperm (Lane 3) but detected in the wash (Lane 4). **(B-C) **YP2 immunoreactivity was detected on both nucleated **(B) **and anucleated **(C) **spermatozoa from the UVD lumen. **(D-E) **The staining was absent in both types of spermatozoa from the seminal vesicles. Thread-like sperm nuclei aligned in nucleated bundle in **(B) **and **(D) **are stained blue with Hoechst 33258. Bar = 20 μm.

Our data indicate that YP2 protein is produced in the cyst cells and is abundantly present in the UVD seminal fluid. To determine whether YP2 become associated with spermatozoa in the UVD lumen, sperm from this compartment was centrifuged and the pellet washed several times. Proteins from sperm and supernatant were separated on the SDS PAGE gel, blotted, and probed with YP2 antibody. Immunoreactive protein of 75 kDa was detected in both seminal fluid and sperm sample (Figure 8A). This protein was dissociated from spermatozoa by washing the sperm fraction with 1 M NaCl. After this high-salt treatment, YP2 was detected in the wash, but was no longer present in the sperm fraction (Figure 8A). These data suggest that YP2 associates with the surface of spermatozoa in the UVD lumen.

To confirm this and to determine spatial distribution of YP2 protein on spermatozoa, we incubated sperm bundles isolated from the UVD lumen with YP2 antibody. YP2 appeared to coat the entire sperm cell surface in both nucleated and anucleated bundles (Figure 8B–C). YP2 was not detected on sperm bundles that moved to the seminal vesicles (Figure 8D–E). Taken together, this data suggest that YP2 associates with the surface of spermatozoa in a transient manner.

## Discussion

Our search for candidate glycoproteins involved in sperm maturation unexpectedly revealed the presence of the yolk protein YP2 in the upper vas deferens (UVD) of male *S. littoralis*. This protein – along with other yolk components, which accumulate in oocyte and provision developing embryo – was believed to be female specific. Previous studies in the moth, *P. interpunctella *have shown that YP2 is produced by the follicle cells and secreted into the oocyte, giving rise to early yolk spheres, which are formed prior to the uptake of other YPs from the fat body [19,24]. Our data suggest that YP2 serves as yolk constituent also in *S. littoralis*. At the same time, we provide two lines of evidence demonstrating that YP2 is also produced in male gonads. First, we cloned *yp2 *cDNA fragment using *S. littoralis *ovaries and detected *yp2 *mRNA in the sperm bundles within the testis. Second, antibody specific for YP2 protein from another moth, *P. interpunctella *detected this protein in the testis of both *P. interpunctella *and *S. littoralis *(Figure 2 and 5). Thus, we propose that the same YP2 protein is produced in the female and male gonads of these two moth species. Further investigation focused on *S. littoralis *because males of this species are substantially larger and produce copious amount of sperm, which facilitated determination of the fate of YP2 in the male reproductive system.

We initially identified YP2 in the seminal fluid bathing sperm released into the UVD, and then showed that the source of YP2 is in the testes. Abundant immunoreactive material was detected in the homogenates of the testes with the molecular weight slightly higher than in seminal fluid. This result suggests that larger precursor of YP2 protein may be produced in the testes and undergo proteolytic cleavage or other modifications upon release into the seminal fluid. In support of this interpretation, Western blot from *P. interpunctella *testis-UVD complexes also showed multipleYP2 species of similar size as in *S. littoralis*. ICC with YP2 antibody on sectioned testes of *S. littoralis *revealed strong immunostaining in the cyst cells, which envelope bundles of spermatozoa until their release from testis. Cyst cells of both nucleated and anucleated sperm bundles were stained with YP2 antibody, while spermatozoa inside the bundles did not appear to be stained.

What might be the role of YP2 protein in the male? The cyst cells in males are developmentally homologous to the follicle cells in females in that both are somatic cells directly associated with developing germline cells. In the follicle cells, YP2 protein is produced as a minor yolk constituent, which is secreted and taken up by the oocyte [19,24]. We show here that in the male, this protein is released into the UVD and becomes associated with sperm. The YP2 behaves as peripheral sperm membrane protein since it can be removed from sperm by treatment with high salt. Previous electron microscopy studies suggested that moth spermatozoa acquire an external coat in the UVD [5,12,13]; however, its molecular identity has not been elucidated. Data reported here suggest that YP2 protein may constitute a component of the sperm coat. Mammalian sperm is known to be coated with many peripheral glycoproteins, which are thought to be involved in the maturation of spermatozoa [9,25]. Our study identified novel sperm coat protein in insects; remarkably, it is a protein that was considered female-specific along with other yolk components.

Our data suggest that the association of YP2 with sperm is of a transient nature. YP coating is detected on sperm present in the UVD; however, it is not detected on sperm translocated to lower parts of the reproductive tract. There are at least two possible explanations for transient YP2 detection on sperm. First, YP2 may persist on sperm but the antigenic regions could be either removed, or become inaccessible to the antibody. Such modifications are known to occur in mammalian sperm surface proteins during sperm passage through the reproductive tract; they may involve transmembrane carbohydrate modifying enzymes, which are known to function in both mammalian and insect sperm [26,27]. The alternative explanation for the absence of YP2 signal on sperm translocated to lower parts of the reproductive tract is that YP2 may dissociate from spermatozoa surface and may be taken up by the epithelial cells forming the UVD wall. We detected YP2-immunoreactive material in these cells by both Western and ICC; however, RT-PCR showed that *yp2 *gene is not expressed in the UVD cells. When UVDs were isolated from the testis by a ligature, there was no YP2 immunoreactivity in the epithelial cells. Taken together, these data suggest that YP2 may be secondarily absorbed from the seminal fluid by the UVD epithelial cells. This scenario is similar to findings in mammals where some proteins secreted by the Sertoli cells into the seminal fluid are reabsorbed by epithelial cells of the epidydimis [11].

In addition – or instead of – yolk proteins produced in the follicular cells, such as YP2, many invertebrate and vertebrate females produce yolk precursors belonging to vitellogenin family, which are synthesized in the fat body or liver [2]. In general, the expression of all yolk protein precursors is restricted to females; however, vitellogenins can be experimentally induced in male insect fat body or vertebrate liver by application of female sex hormones [20,28-30]. Interestingly, vitellogenin gene appear to be expressed in the fat body of honeybee males (drones) without any hormonal treatment [31]. In addition, major yolk protein of the sea urchin is present in both males and females [32,33]; however, this protein is not related to vitellogenins of other animals. Taken together, these reports suggest caution in considering yolk proteins as female specific, especially in view of our data, which provide strong evidence linking yolk protein precursor with processing of sperm. While specific roles of YP2 in the male remain to be elucidated, our data uncovered a remarkable and unexpected commonality in the maturation of eggs and sperm.

## Conclusion

Yolk protein precursors accumulate in oocytes and provision developing embryos. We show here additional male-specific role for one of the yolk protein precursor, YP2. This protein is produced in testes of intact males, is released into the seminal fluid, and becomes attached to spermatozoa. These novel findings suggest that male cyst cells and female follicle cells may use the same protein to fulfill different functions in the development of gametes in each sex.

## Methods

This research was performed on the moth *Spodoptera littoralis *(Noctuidae) maintained at 26°C, in a photoperiod of 16 h light and 8 h darkness (LD 16:8) on artificial diet [34]. Some experiments were performed on *Plodia interpunctella *(Pyralidae) maintained on whole-wheat flour. Males for experiments were separated from females as pupae, and used as 2-day-old adults. As sperm release begins prior to adult eclosion, males of this age are sexually active. Unless otherwise indicated, experiments were performed on males collected at four hours after lights-off, which is designated as Zeitgeber Time (ZT) 16. There is maximal accumulation of sperm in the UVD at this time [14].

### Analysis of proteins from seminal fluid using 2D electrophoresis

Reproductive systems were dissected from males at four hours after lights-on (ZT4) and at four hours after lights-off (ZT16). Testis-UVD complexes were transferred into Grace's medium (GIBCO BRL). To obtain samples of seminal fluid, content of 50 UVD was released into 50 μl of medium. Each sample was centrifuged at 4,000 g for 6 min to pellet the sperm, and the supernatant (seminal fluid) was removed to another tube and mixed 20:1 with Proteinase Inhibitor Cocktail (PIC, Sigma-Aldrich). Bio-Rad Protein Assay (Bio-Rad, USA) was used for quantitation of total protein in each sample. Proteins were purified with the 2-D Clean-Up Kit (Amersham-Bioscience, USA), and precipitated with acetone for 2 h at -20°C. The samples were centrifuged for 15 min at 4°C and 14,000 g, and the pellet was dried for 30 min at room temperature. The first electrophoretic direction was performed using set linear pH 3–10 gradient strips (Amersham-Bioscience). The separation conditions were: 150 V for 1 h, a gradient ramp from 300 V to 1500 V for 1.5 h, 1500 V for 14–20 h. The second direction was performed in 10% acrylamide gels. The separation by mass was performed at an initial current density of 30 mA for 10 min, then at 50 mA for 10 min, and 100 mA until completion. Full range Rainbow recombinant protein molecular weight marker (Amersham-Bioscience) and CandyCane Glycoprotein Molecular Weight Standards (Invitrogen, USA) were run in the outer lanes of the gel.

### Detection and sequencing of glycoproteins from the seminal fluid

To stain glycoproteins in the gel, the Pro-Q Emerald 300 Staining Glycoprotein Gel and Blot Stain Kit (Invitrogen, USA) was used according to the manufacturer's instructions. The images were analyzed using a Gel Doc 2000 (Biorad, USA). Prominent glycoproteins were cut out from the gels and digested with trypsin. Peptide sequences were determined with Q-TOF mass spectrometer (Micromass) combined with the nano-HPLC system. The sequences were analyzed using the MassLynx 3.5 package against BLAST protein database for short, nearly exact matches.

### Cloning of yp2 cDNA from *S. littoralis *ovaries

The published sequences of the yolk polypeptide 2 from *Plodia interpunctella *[GenePept:AAC62229], yolk protein 2 from *Galleria mellonella *[GenPept:AAB09081], and egg specific protein of *Bombyx mori *[GenPept:BAA02091] were compared for conserved regions using clustalW software [22]; these regions were used to design degenerate primers. A set of primers Eyp2F1, 5'-TAYCARTAYCCNGTNGARGARCA-3' and Eyp2R4, 5'-TGGCTNGGNAAYGTNCGNGGNAA-3' resulted in amplification of the expected band. Total RNA from ovaries of 2-day old *S. littoralis *females was extracted with RNA isolation reagent PureZOL (Biorad) and treated with RQ1 DNase I (Promega). First strand synthesis was performed with AMV Reverse Transcriptase XL (Takara Bio, Japan), using 5 pmol of Oligo(dT) (Qiagen GmbH, Germany) and 15 μg of total RNA.

Touchdown PCR was performed as follows: 94°C for 5 min followed by 10 cycles: 94°C for 1 min, 41°C for 2 min with ramp 7°C/min to 72°C and 3 min at 72°C, than 20 cycles: 94°C for 1 min, 41°C for 2 min, 72°C followed by final extension at 72°C for 15 min Generated PCR fragment ~240 bp was purified and ligated into the pCR II TOPO vector using the pCR TOPO^® ^TA cloning kit (Invitrogen). Its sequence was determined by sequencing with M13-reverse primer (AGOWA GmbH, Germany) and compared for similarity to yolk protein coding sequences from other species using BLAST. To elongate the fragment of the putative *S. littoralis yp2 *gene, the rapid amplification of cDNA 3' end (3'-RACE) was used with Takara's 3'-Full RACE Core Set (Takara Bio) and the following gene specific primer: GTTGTTTTCCTGATGCACG. The 3'RACE identified 1201-bp fragment; the sequence was submitted [Genbank:EU368829] and used to design specific primers for analysis of *yp2 *gene expression in different tissues of *S. littoralis*.

To study expression of *yp2*, we collected *S. littoralis *testis, sperm bundles, ovaries, and male and female carcasses without gonads at ZT16. Isolation and purification of total RNA from these samples and RT-PCR reactions were identical as described above. Two sets of primers were used: for *yp2 *forward primer YP2F1, 5'-CTGGCTACGACGTATGGCTCGGTA-3', reverse primer YP2R1, 5'-CTTAGGAGGCTCTTCCGAACCCA-3' and for *28S r-RNA *gene (RNA loading control): forward 28S1F, 5'-GAAAGAAGCCCAGCACTGAAT-3' and reverse primer 28S1R, 5'-CACTCTCAAGCAACCCGACTC-3'. PCR resulted in two expected products of 660 bps for *yp2 *and 199 bps for *28S r-RNA*; they were sequenced for verification.

### Western blotting

Total protein extractions from various tissues of *S. littoralis *were performed as described for 2D electrophoresis except 2-D Clean-Up Kit was not used. Hemolymph was centrifuged at 4,000 g to remove hemocytes. Equal amounts of total protein (1 OD) based on Bio-Rad Protein Assay quantitation were loaded onto 10% SDS-PAGE Precast Gels (Biorad), separated, and transferred to PVDF Immobilon-FL membrane (Millipore). Protein Kaleidoscope standards (Biorad) and TriChromRanger Marker (Pierce) were used. Blots were probed overnight at 4°C with rabbit antiserum against *Plodia interpunctella *Yolk Protein 2 (YP2) [18] diluted 1:3000 in Odyssey Blocking Buffer (LI-COR Biosciences) containing 0.1% Tween 20 (v/v%). Following four washes in 10 mM PBS pH 7.5 containing 0.05% Tween 20 (PBST) blots were incubated for 1 h at room temperature in IRDye 680 Goat Anti-Rabbit IgG (LI-COR Bioscience, USA) diluted 1:20000 in Odyssey Blocking Buffer supplemented with 0.05% Tween 20 and 0.01% SDS. Membranes were then washed four times in PBST, dried and imaged with the Odyssey Infrared Imager (LI-COR Biosciences) using 700 nm channel. In the initial experiments, blots of seminal fluid were incubated also with the antibodies against YP1, YP3, and YP4 from *P. interpunctella *[18] (all tested in three dilutions: 1:500, 1:1000, 1:3000) and with antibody detecting YP1-3 of *Drosophila melanogaster *[20] diluted 1:1000.

### Immunocytochemistry

Organs of *S. littoralis *were fixed in 4% paraformaldehyde in 0.1 M sodium-phosphate-buffered saline pH 7.5 (PBS) at 4°C for 2 h. The testes with attached UVD and seminal vesicles were dehydrated through an increasing ethanol series, embedded in paraplast (McCormick Scientific), and cut into 6 μm thick sections. Re-hydrated sections of male reproductive organs and fixed whole ovarioles were washed in PBS and blocked for 1 h in 5% normal goat serum (NGS), 0.1% bovine serum albumin (BSA) in PBS containing 0.3% Triton X-100 (TX-100). Specimens were incubated for 12 h at 4°C *Plodia interpunctella *YP2 antibody [18] diluted 1:1000 in PBS containing 0.1% BSA, 3% NGS and 0.03% TX-100 (PBST-NGS). Alexa Fluor 594 Fab'2 fragment of goat anti-rabbit IgG (Invitrogen) diluted 1:1000 in PBST-NGS was applied to slides for 1 h at room temperature. Hoechst 33258 (Invitrogen) diluted 1:10000 in PBS was used to stain cell nuclei. Both tissue sections and ovarioles were mounted in Fluoromount-G (Southern Biotech). The testis-UVD slides were examined under DMBR Leica fluorescence microscope equipped with SPOT camera (Diagnostic Instruments). Whole-mounted ovarioles were examined in Confocal Laser Scanning Microscopy Zeiss LSM 510 Meta. For immunostaining of sperm, spermatozoa from the UVD were rinsed in PBS and air-dried on the glass slides, followed by fixation in 4% paraformaldehyde/PBS pH 7.4. ICC was performed as described above, except that YP2 antibody was diluted 1:500. To verify the specificity of anti-YP2 we used as negative control antibody preabsorbed with *P. intepunctella *ovarioles (2 μl of serum was diluted 1:100 in PBST-NGS and incubated overnight with paraformaldehyde fixed ovarioles from 10 newly emerged adult females; then ovarioles were removed and the antibody solution was adjusted to concentration 1:500 or 1:1000 and used for ICC). To prevent the release of sperm and other material from the testes prior to ICC, incision was made in the cuticle of CO_2_-anesthetised male, reproductive system was briefly exposed, and a thin silk thread was tied between the testes and the UVD. Sham operated males were treated identically except ligature was not applied. Reproductive systems from experimental and control sham-operated males were dissected after 8 hours, fixed, sectioned, and stained with YP2 antibody as described above.

### Separation of peripheral membrane proteins from spermatozoa

Spermatozoa collected from 50 UVDs were washed in Grace's medium and incubated with gentle agitation for 1 h at room temperature in 0.5 ml of 0.1 M PBS (pH 7.5) containing 1 M NaCl. After centrifugation, supernatant proteins were precipitated with acetone for 24 h at 20°C. After 1 h centrifugation at 15000 g, protein pellet was dried, re-suspended in protein loading buffer, and analyzed by Western blot along with the sample of centrifuged sperm.

## Abbreviations

UVD: upper vas deferens; ZT: Zeitgeber Time; YP2: yolk protein 2; ICC: Immunocytochemistry; RACE: rapid amplification of cDNA ends; RT-PCR: reverse transcriptase-polymerase chain reaction; bp: nucleotide base pairs; kb: kilo base pairs; nt: nucleotide.

## Authors' contributions

PB, JK and JMG conceived and designed the experiments. PB, JK and EJ performed the experiments and analyzed the data. JMG wrote the paper with help from PB and JK.
